# Effectiveness of Acceptance and Commitment Therapy (ACT) in Patient with Cardiovascular Disease: A Systematic Review

**DOI:** 10.3390/healthcare13151831

**Published:** 2025-07-27

**Authors:** Alessandro Grimaldi, Isabella Veneziani, Laura Culicetto, Angelo Quartarone, Rocco Salvatore Calabrò, Desirèe Latella

**Affiliations:** 1 Department of Nervous System and Behavioural Sciences, Psychology Section, University of Pavia, Piazza Botta, 11, 27100 Pavia, Italy; alessandrogrimaldi1997@gmail.com (A.G.); venezianiisabella@gmail.com (I.V.); 2 IRCCS Centro Neurolesi “Bonino-Pulejo”, S.S. 113 Via Palermo, C. da Casazza, 98124 Messina, Italy; angelo.quartarone@irccsme.it (A.Q.); roccos.calabro@irccsme.it (R.S.C.); desiree.latella@irccsme.it (D.L.)

**Keywords:** acceptance and commitment therapy, psychological flexibility, emotional regulation, cardiovascular disease, chronic illness, mental health, quality of life, rehabilitation

## Abstract

**Background/Objectives:** Cardiovascular diseases (CVDs) encompass a wide range of heart and vascular conditions and remain the leading cause of death worldwide. Acceptance and Commitment Therapy (ACT) is a psychotherapeutic approach that integrates acceptance, mindfulness, and commitment to value-based actions. This systematic review aims to explore the current evidence on the potential role of ACT interventions in supporting psychological well-being among individuals with CVDs. **Methods:** A systematic review was conducted in accordance with PRISMA guidelines. A search of the literature was conducted through Scopus, PubMed, Web of Science, Cochrane, and PsycINFO databases. Six studies met the inclusion criteria. **Results:** The reviewed studies suggest that ACT may promote psychological flexibility, emotion regulation, and self-care behaviors in patients with CVDs. Reported outcomes include improved mindfulness, reduced distress, and enhanced quality of life. However, the evidence base is limited in both size and methodological rigor, with included studies varying in design and population. **Conclusions:** While preliminary findings indicate that ACT shows promise in addressing psychological aspects of CVDs, the current evidence remains insufficient to draw definitive conclusions. Further high-quality, large-scale studies are needed to evaluate the effectiveness and clinical applicability of ACT in cardiovascular populations.

## 1. Introduction

Cardiovascular diseases (CVDs) encompass a broad spectrum of disorders including ischemic heart disease, stroke, heart failure, peripheral arterial disease, and various other cardiac and vascular conditions [1]. CVDs represent the leading cause of death worldwide, claiming an estimated 17.9 million lives annually and significantly impacting overall quality of life (QoL) [2,3]. Despite an overall decrease in global mortality rates, deaths attributed to CVDs, particularly ischemic heart disease and stroke, have risen by a concerning 21.1% between 2007 and 2017 [3,4]. Currently, heart attacks and strokes are responsible for 80% of CVD deaths, with a particular impact on younger individuals, as one-third of these deaths occur prematurely in those under 70 years old.

### 1.1. Epidemiological Burden in Italy

In Italy, CVDs are the leading cause of mortality, accounting for 44% of all deaths [5]. Moreover, cardiovascular disability affects a considerable segment of the population, with a prevalence estimated at 4.4 per 1000 inhabitants [5]. These data highlight the urgent need for innovative therapeutic approaches and effective preventive strategies to address this growing public health challenge.

### 1.2. Stress as a Cardiovascular Risk Factor

Recent studies have identified stress as a significant and newly recognized risk factor for CVDs. Prospective data from large-scale cohort studies confirm a strong association between stress and the incidence of stroke, coronary heart disease, atrial fibrillation, and other cardiovascular manifestations [3,4]. While workplace stress and social isolation are the most widely studied triggers, other psychosocial stressors—such as marital problems, caregiving responsibilities, and bereavement—also contribute to cardiovascular risk. Stress-related emotional disturbances, including depression [5,6], anxiety [7], psychological distress [8], emotional exhaustion [9], and post-traumatic stress disorder (PTSD) [10], are not merely causes of stress, but often emerge as its consequences.

### 1.3. The Interplay Between Depression and CVDs

In healthy individuals, depression increases the risk of coronary artery disease by 1.5 to 2.0 times, while in patients with pre-existing coronary disease, it raises the risk of myocardial infarction by 1.5 to 4.5 times [11,12]. The coexistence of depression and CVDs is associated with worse clinical outcomes and elevated healthcare costs. Conditions such as hypertension and coronary heart disease are frequently accompanied by depression, emphasizing the strong bidirectional link between mental health and cardiovascular risk. Consequently, the Italian National Health Service (NHS) has prioritized prevention and provided access to innovative cardiovascular treatments. A truly effective preventive strategy should integrate interventions targeting lifestyle, risk factor modification, disease management, and rehabilitation [13,14].

Nonetheless, significant challenges persist, ranging from the insufficient implementation of preventive strategies by physicians to issues such as patient mistrust and the spread of misinformation, including fake news, regarding medications and the adoption of healthy lifestyles. In general, it is crucial for every patient to actively participate in understanding and managing key risk factors associated with CVDs, encompassing aspects like smoking, physical activity, diet, and levels of blood cholesterol, glucose, and pressure.

Effective CVD prevention requires active patient participation in understanding and managing key risk factors like smoking, diet, physical activity, and blood pressure, cholesterol, and glucose levels [15,16]. However, the lack of internationally standardized guidelines for risk assessment and interventions remains a challenge. For elderly patients, prevention programs face additional challenges due to emotional changes, altered body perception, and diminished future expectations [17,18]. CVDs can also impair cognitive function in older adults, potentially leading to somatic dysfunction and contributing to geriatric depression.

Recognizing this association is crucial for optimizing treatment strategies in CVD patients.

Research suggests that cognitive behavioral therapy (CBT) techniques, particularly Acceptance and Commitment Therapy (ACT), can effectively motivate older adults to adopt healthier lifestyles [19,20]. ACT is a form of CBT that uses mindfulness, acceptance, and commitment to enhance psychological flexibility. This approach differs from traditional CBT by focusing on normal human cognition rather than specific deficits [21]. ACT offers several potential benefits for CVD management. By incorporating mindfulness and acceptance techniques, this approach aids individuals in coping with stress, a major risk factor for CVDs [21], thereby promoting emotional acceptance and improving mental health [22]. Additionally, ACT emphasizes values-based actions, potentially leading to healthier lifestyle choices [23], and enhances psychological flexibility, enabling individuals to navigate life challenges more effectively, which may reduce their negative impact on cardiovascular health [24]. Furthermore, ACT may improve adherence to medication and self-management programs [25]. ACT’s different focus improves well-being and QoL of those involved by advanced progressive illness, including caregivers and healthcare personnel. The ACT model consists of six interdependent and overlapping processes. The first is acceptance, as creating a space for difficult thoughts and emotions rather than suppression or avoidance them. Defusion is the detachment from negative thoughts and emotions to reduce their influence on cognition. Contact with the present moment involves tuning into what is happening in the environment right now maintaining flexible awareness hic et nunc rather than being influenced by the past or future. Self as context is a viewpoint from which we can observe thoughts and feelings, and a psychological space in which those thoughts and feelings can move. It is a place from which we can observe our experience without being caught up in it. Values are the vital and personally meaningful aspects of life. Clarifying values allows the creation of behavioral change. The last element, committed action, involves engaging in patterns of effective action that are guided by core values. These processes can be broadly classified into two categories: (1) mindfulness and acceptance, and (2) commitment and behavior change [26]. ACT is a collaborative process in which the therapist and the client work together to promote awareness, psychological flexibility, and behavioral change towards a more satisfying and meaningful life [27]. A significant advantage of ACT compared to other therapeutic approaches is its integration of motivational and cognitive elements, leading to more enduring treatment outcomes [28]. There is a rapidly growing evidence base for the efficacy of ACT in the treatment of a broad range of disorders and conditions, with recent systematic reviews describing its potential value for CDVs [29,30,31,32,33].

This systematic review aims to evaluate the current body of research on the effectiveness of ACT interventions in managing and preventing CVDs.

## 2. Materials and Methods

**Figure 1:**
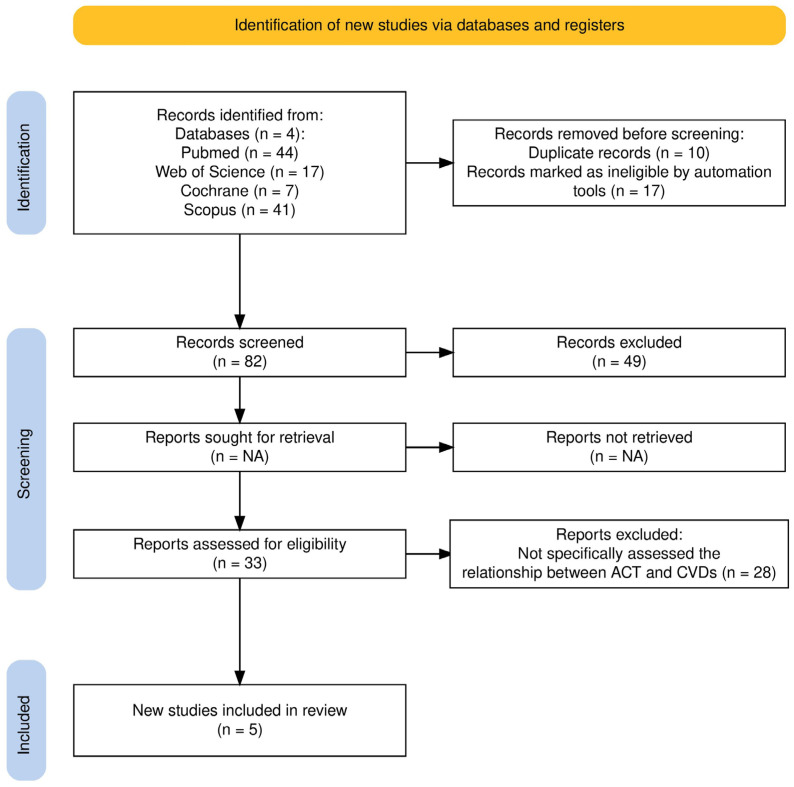
Prisma flow diagram for research strategy.

This systematic review was conducted and reported in accordance with the Preferred Reporting Items for Systematic Review and Meta-Analyses (PRISMA) (see Figure 1) [34] See Supplementary Materials for the PRISMA 2020 Checklist. A protocol for this review was registered on the Open Science Framework (OSF), registration code/number: DOI 10.17605/OSF.IO/TCFPS, registration date: 13 March 2024.

### 2.1. Search Strategy

The studies were identified by searching in Scopus, PubMed, Web of Science, Cochrane, and Psychinfo databases. All the studies fulfilling our selected criteria were evaluated for possible inclusion. The search combined the following terms: (“acceptance and commitment therapy” [MeSH Terms] OR (“acceptance” [All Fields] AND “commitment” [All Fields] AND “therapy” [All Fields]) OR “acceptance and commitment therapy” [All Fields]) AND (“cardiovascular diseases” [MeSH Terms] OR (“cardiovascular” [All Fields] AND “diseases” [All Fields]) OR “cardiovascular diseases” [All Fields] OR (“cardiovascular” [All Fields] AND “disease” [All Fields]) OR “cardiovascular disease” [All Fields]). The search terms were identified for title and abstract. After duplicates had been removed, all articles were evaluated based on title and abstract.

This research was not restricted by the year of publication for the articles considered.

Inclusion criteria were (i) articles that enrolled human subjects, (ii) experiments that examined ACT in CVDs patients, and (iii) articles in the English language only.

Exclusion criteria were (i) reviews and meta-analyses and (ii) duplicated studies.

The literature search was conducted between 15 February and 10 April 2024.

### 2.2. Study Selection

A total of 109 articles were identified through database searches. In total, 10 articles duplicated were deleted, 17 reviews were removed, 42 studies were removed for title screening, 4 for abstracts, and 28 articles were removed for text screening (Figure 1). In this systematic review, we considered a total of 6 articles about the relationship between ACT and CVDs. To minimize bias and ensure a robust selection process, two authors (A.G. and I.V.) independently reviewed and extracted data from the studies. Any discrepancies were resolved through collaborative discussion and consultation with a third author (D.L.). This multi-step approach guaranteed that at least three researchers independently assessed each article. In cases of persistent disagreement, all authors were involved in the final decision. Data extraction relied solely on the full text of the articles.

### 2.3. Data Extraction and Analysis

After finalizing the selection through full-text review, data were extracted from the included studies and compiled into a table using Microsoft Excel 2021. The extracted information included key study characteristics: title, lead author, publication year, aims, design, sample size, participant demographics, intervention and control details, baseline measures, outcome types with assessment points, results, and key conclusions. We also extracted data on reported effect sizes and power analyses. Cases where such data were not provided by the original authors have been explicitly noted in in the corresponding table. Additionally, inter-rater reliability between the two reviewers (I.V. and A.G.) was assessed using the kappa statistic. The kappa score, exceeding the 0.61 threshold for substantial agreement, indicated excellent concordance between the reviewers. This high score underscores a robust data extraction process with a significant level of agreement. Given the limited number and heterogeneity of the included studies (randomized controlled trials, observational, and qualitative designs), we opted for a combined synthesis approach to provide an exploratory overview of the emerging evidence on ACT in cardiovascular populations. This strategy prioritizes hypothesis generation over definitive conclusions, as stratification by design type was not feasible due to the small number of studies in each category.

### 2.4. Assess Quality of Included Studies’ Risk of Bias

The risk of bias in randomized controlled trials (RCTs) was assessed using a revised Cochrane risk of bias (RoB 2) tool [34], which comprises five domains: (i) bias arising from the randomization process, (ii) bias due to deviations from the intended intervention, (iii) bias due to missing outcome data, (iv) bias in the measurement of the outcome, and (v) bias in the selection of the reported result. Furthermore, the risk of bias in non-randomized studies of exposures (ROBINS-E) tool [35] evaluates seven key domains that can introduce bias: (i) bias due to confounding, (ii) bias arising from measurement of the exposure, (iii) bias in selection of participants into the study (or into the analysis), (iv) bias due to post-exposure interventions, (v) bias due to missing data, (vi) bias arising from measurement of the outcome, and (vii) bias in the selection of the reported results (see [36]).

## 3. Results

### 3.1. Synthesis of Evidence

We conducted a systematic review to evaluate the current body of research on the effectiveness of ACT interventions in managing cardiovascular diseases (CVDs). A total of six studies were included (Table 1); two focused on exploring the mechanisms of ACT, while the remaining four assessed patient outcomes. These studies encompassed a range of methodological designs, including randomized controlled trials, quasi-experimental studies, observational research, and qualitative analyses. Given the heterogeneity of the included designs and the limited number of studies, we opted to synthesize findings across methodologies to provide a broad overview of the emerging evidence. However, we acknowledge that this approach may weaken the internal validity of the conclusions due to the lack of stratification by design type. Therefore, the results should be interpreted as exploratory and hypothesis-generating, rather than definitive. Future reviews with larger datasets may benefit from stratifying findings by study design to enhance interpretability and methodological rigor.

For further details on study characteristics, outcomes, and ACT intervention protocols, see Table 1 and Table 2.

### 3.2. Key Findings from Included Studies

The studies reviewed clarified the potential effectiveness of mechanisms of ACT such as present-moment focus, mindfulness, and emotion regulation skills. Although not directly assessed in terms of physiological parameters in the included studies, these processes have been theoretically linked to autonomic regulation, particularly parasympathetic functioning. This association is hypothesized to contribute to the perceived therapeutic value of ACT by fostering a supportive and emotionally regulated environment during treatment sessions. Similarly, Al-Hammouri et al. [37] explored the application of ACT in promoting self-care behaviors among individuals with heart failure. Their study highlighted the essential role of mindfulness, cognitive fusion, and committed action in the maintenance of self-care practices. Notably, committed action positively affects self-care behavior, especially among individuals with lower levels of mindfulness and cognitive fusion. These results emphasize the importance of evidence-based interventions in enhancing self-care practices among heart failure patients.

Studies investigating patient outcomes [39,40,41,42] demonstrated the positive effects of ACT interventions across various cardiovascular conditions. Ghahnaviyeh’s study [38] evidences the effectiveness of ACT intervention in ameliorating the QoL in patients with myocardial infarction. ACT approach focuses on functional processes underlying impaired behavioral manifestations rather than on a disorder’s characteristic symptoms developing functional behavioral patterns. Instead of focusing on reducing the symptoms, the therapist seeks to improve the patient’s overall QoL with his cooperation. This method improves both mental and physical health. ACT intervention allows these patients to clarify their values and goals, focusing on things they can change rather than on things they cannot. In patients with myocardial infarction, basic activities such as sitting or lying down to rest, going out, walking, climbing stairs, sleeping and shortness of breath are challenges. As a result, patients learn that, in alignment with their beliefs, it is preferable to avoid the negative/intrusive thoughts and the subsequent effects. In addition, Sheibani et al. (2019) [39] examined the effects of ACT on emotion regulation and self-control strategies in patients with coronary heart disease. Their findings indicated that group-based ACT sessions help patients choose functional emotion regulation strategies, avoiding dysfunctional ones. Fattahi et al. [40] further supported these findings by demonstrating that ACT improved emotion regulation and self-compassion in cardiovascular patients. They suggest that ACT interventions help patients accept their thoughts and feelings rather than trying to change them, leading to more flexible and compassionate self-awareness. Similarly, Large, Samuel, and Morris’s study [41] suggests that ACT can help stroke survivors accept their condition through reappraisal and cognitive flexibility. However, the authors suggest a tailored approach as each participant had diverse needs.

Overall, these studies provide encouraging evidence for the effectiveness of ACT in improving mental and emotional well-being in patients with CVDs. The studies also shed light on potential mechanisms by which ACT works, including fostering a supportive therapeutic environment, promoting mindfulness and cognitive flexibility, and encouraging acceptance and self-compassion.

### 3.3. Risk of Bias

**Figure 2:**
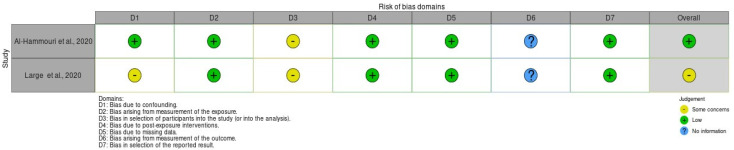
Risk of bias (ROBINS-E) of studies [37,41].

**Figure 3:**
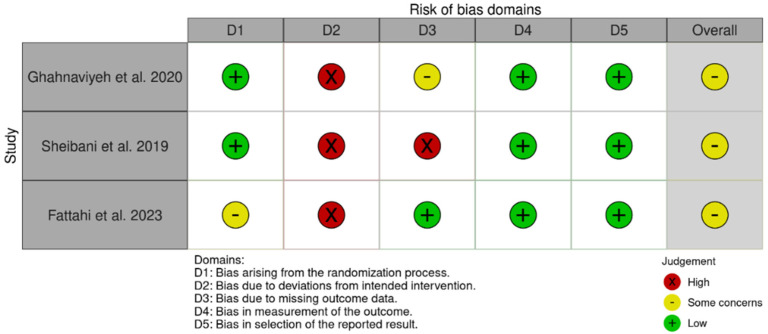
Risk of bias of RCTs (RoB 2) [38,39,40].

The Cochrane risk of bias (RoB 2) tool and the Risk Of Bias In Non-randomized Studies-of Exposures (ROBINS-E) tool were used to assess the risk of bias of the articles included in this review. Figure 2 and Figure 3 show the summary of the risk of bias assessment. Out of the total studies assessed, one study showed some concerns about bias due to confounding [42]. Additionally, two studies displayed some concerns about bias in selection of participants into the studies [37,42]. Moreover, only one study showed some concerns about bias arising from measurement of the outcome [38]. Three studies exhibited high risk of bias to deviations from the intended intervention [39,40,41]. Furthermore, one study showed high risk of bias due to missing outcome data [40] and another some concerns [39]. Only one study displayed some concerns about bias arising from the randomization process [41].

## 4. Discussion

This review provides preliminary evidence suggesting that ACT may support mental and emotional well-being in patients with CVDs. The literature highlights the significant psychological burden associated with these conditions, with patients frequently experiencing fear, anxiety, and depression, which can hinder effective disease management [43]. ACT has been proposed to address these challenges by promoting acceptance and psychological flexibility [44]. Unlike traditional therapies focused on symptom suppression, ACT fosters mindful observation of internal experiences, enabling patients to build resilience through cognitive defusion and values-oriented behavior [45]. This therapeutic process may help patients relate differently to distressing emotions, potentially enhancing emotional regulation and interpersonal functioning. Furthermore, ACT facilitates the identification and pursuit of personal values despite illness-related limitations [46].

Ghahnaviyeh et al. [38] found that ACT significantly improved both physical and mental components of quality of life in myocardial infarction patients, with benefits maintained at a six-month follow-up. Similarly, Fattahi et al. [40] demonstrated a significant long-term reduction in depression, anxiety, and stress, accompanied by improvements in self-compassion and emotion regulation. Effect sizes were strongest for stress (η^2^ = 0.69), followed by suppression, self-compassion, and anxiety. Additionally, the qualitative study by Celano et al. (2016) [42] uniquely captured the experiential recovery process in post-stroke patients. Participants reported that ACT helped them reinterpret disability-related limitations and regain a sense of agency. Thematic analysis revealed that psychological flexibility fostered by ACT contributed to improved coping, emotional regulation, and acceptance of a changed reality [42]. Notably, earlier systematic reviews by Dindo et al. (2017) [43] and McPhillips et al. (2019) [44] included 12 and 15 studies, respectively, but focused more broadly on chronic illness or predated several recent contributions now included in the present review. Similar lines of inquiry have also been explored by other authors, further supporting the relevance of this topic [43,44,45,46,47]. By integrating newer trials with longitudinal designs and diverse CVD populations (e.g., post-stroke, heart failure), this synthesis offers enhanced depth and contemporary relevance. Nevertheless, the limited number of included studies (n = 5) and their methodological diversity (three RCTs, one observational, one qualitative) constrain the strength and generalizability of our conclusions.

Broader meta-analytic efforts remain necessary to quantify pooled effects across outcomes and subgroups [48]. While this review focuses on ACT, it is important to acknowledge other psychological interventions effective in CVD populations. Traditional cognitive behavioral therapy (CBT), for instance, emphasizes cognitive restructuring and has been widely used to reduce psychological distress and improve treatment adherence. Mindfulness-Based Stress Reduction (MBSR) and psychoeducational programs have also shown promise, particularly in enhancing emotional regulation and reducing stress. Compared to these approaches, ACT centers more explicitly on acceptance and values-driven action rather than symptom elimination. Although some studies suggest ACT may offer unique advantages for patients coping with chronic illness, direct head-to-head comparisons remain limited. Future research should aim to contrast ACT with these established interventions to determine its relative efficacy and suitability for different patient profiles.

ACT may also offer added value by equipping individuals with effective emotion regulation skills, which are critical for managing the psychological impact of CVD. Through mindfulness and cognitive defusion, patients learn to observe thoughts and feelings with greater clarity and reduced reactivity. This, in turn, lessens the intensity of negative emotions and supports adaptive coping [49,50]. The importance of emotion regulation is corroborated by studies such as [40,41], which report concurrent improvements in self-compassion and psychological well-being. These psychological benefits may, according to some hypotheses, positively influence cardiovascular outcomes, given the established link between emotional distress and cardiac prognosis [47]. Overall, the findings reviewed here indicate that ACT may contribute to reduced psychological distress, enhanced emotion regulation, and improved self-compassion in CVD patients [37,38,39,40,41,42]. For example, ACT has been shown to help stroke survivors accept their new reality, reinforcing its broader potential in chronic care contexts [42]. By complementing traditional medical treatments with a psychological framework rooted in flexibility and meaning, ACT may promote active engagement in health management [39]. Some studies highlight the need for tailored interventions, suggesting that ACT protocols should be adapted to specific CVD subgroups. Furthermore, future research should explore digital delivery platforms (e.g., mobile-based ACT) and assess the cost-effectiveness of these approaches. As emphasized across several studies, interventions that promote acceptance and values-guided behavior may work synergistically with standard care by targeting psychosocial determinants of cardiovascular prognosis.

Finally, ACT has been associated with benefits beyond mental health. Some studies report improved cognitive function, possibly related to enhanced psychological flexibility and present-moment focus [49]. ACT may also promote greater adherence to treatment plans by helping patients accept their condition and commit to valued actions [50]. By encouraging patients to identify what truly matters to them and focus on controllable aspects of life, ACT fosters a sense of agency, even in the presence of functional limitations [50. Ultimately, ACT may enhance quality of life for individuals with CVD by supporting psychological adjustment and personal growth in the context of chronic disease [50]. While these outcomes are promising, they must be interpreted with caution. Given the heterogeneity of included studies, the present findings remain exploratory. Future systematic reviews should aim for clearer stratification by study design and larger sample sizes to support stronger, evidence-based conclusions.

In particular, future meta-analytic efforts will be essential to quantify the pooled effect of ACT interventions on psychological and health-related outcomes in CVD populations. This will require more homogeneous data across studies in terms of design and measurement. The methodological heterogeneity of the included studies—encompassing randomized controlled trials, cross-sectional designs, and one qualitative investigation—reflects the exploratory nature of the field. We chose to include all relevant study types to ensure a comprehensive mapping of the existing literature. However, this approach inevitably limits direct comparisons between outcomes and reduces the possibility of synthesis across designs. Another important limitation is the incomplete reporting of effect sizes and power analysis across studies, which reduces the ability to compare the efficacy of ACT interventions quantitatively. We emphasize that the small number of available studies significantly limits the generalizability of our findings, and this review should be interpreted as an initial synthesis of a still-developing area of research.

### 4.1. Limitations

Although this systematic review provides valuable insights, there are some limitations that need to be considered. First, only five studies met the inclusion criteria, which limits the statistical and conceptual power of the review. Among these, three were randomized controlled trials, two were observational studies, and one was qualitative. Overall, the studies included small sample sizes, ranging from 12 to 165 participants, with the largest being an exploratory cross-sectional study. This limited number and heterogeneity of studies may impact the generalizability of our findings. However, this reflects the relatively recent application of ACT in the context of CVD management, where evidence is still emerging. Further high-quality, large-scale clinical trials are needed to better evaluate the efficacy, feasibility, and implementation of ACT in patients with CVDs.

### 4.2. Future Directions

Looking ahead, the future of ACT for CVD management holds significant promise. We can expect continued research efforts to refine ACT protocols, tailoring them to the unique needs of different CVD patient subgroups. This personalized approach ensures interventions effectively address the challenges faced by individuals with varying conditions and severities. Technological advancements and telehealth hold the potential to revolutionize access to ACT. Digital platforms and mobile applications delivering ACT-based therapy could significantly broaden dissemination, reaching a wider population of CVD patients, including those in underserved or remote areas. Furthermore, fostering collaboration between healthcare providers, researchers, and policymakers is essential to seamlessly integrate ACT into comprehensive CVDs care models. Advocating for reimbursement policies and insurance coverage for psychological interventions like ACT will ensure equitable access to these evidence-based treatments.

As our understanding of the psychological aspects of CVDs deepens, the importance of addressing patients’ emotional and mental well-being alongside physical health becomes increasingly clear. ACT provides a valuable framework for achieving this holistic approach by promoting acceptance, psychological flexibility, and alignment with personal values.

## 5. Conclusions

This systematic review provides preliminary evidence suggesting that ACT may support emotional well-being and psychological flexibility in individuals with heart failure and other cardiovascular conditions. Across the limited number of studies included, ACT interventions appeared to promote self-regulation, improve coping strategies, and reduce psychological distress. However, the heterogeneity in study designs, small sample sizes, and limited methodological robustness significantly constrain the generalizability of these findings. Given these limitations, the current literature does not allow for definitive conclusions about ACT’s clinical effectiveness in CVD populations. Nonetheless, the promising patterns observed highlight the potential of ACT as a complementary approach in cardiovascular care. Future research should prioritize large-scale randomized controlled trials with standardized protocols and long-term follow-up to rigorously assess the efficacy, mechanisms of action, and implementation strategies of ACT in this context.

## Figures and Tables

**Table 1:** Characteristics of the included studies.

Study	Design	Population	Objective	Intervention	Results	Statistical Outcomes
Al-Hammouri et al., 2020 [37]	Exploratory cross-sectional	165 participants with heart failure	Explore the effect of ACT on the self-care behavior of individuals with heart failure.	ACT model for self-care behavior	Mindfulness and committed action play a significant role in self-care behavior for people with heart failure, but cognitive fusion does not have a direct impact.	Significant bivariate correlations (r = 0.18–0.25, *p* < 0.05–0.001); Regression Model 2 F(5,159) = 3.88, *p* < 0.01; significant predictors: mindfulness, committed action; effect size and power: NR
Ghahnaviyeh et al., 2020 [38]	Controlled clinical trial	60 patients with MI randomly divided into EG and CG.	Evaluate the impact of ACT on the psychological flexibility and overall QoL of MI patients.	ACT for EG, no intervention for CG.	EG showed a noteworthy improvement in their QoL and in subscales related to their mental and physical health.	Repeated measures ANOVA: all main effects *p* < 0.001; physical QoL interaction *p* = 0.03; effect size and power: NR
Sheibani et al., 2019 [39]	Quasi-experimental with a pretest-posttest design	30 patients with coronary heart disease randomly divided into EG and CG.	Examine the effect of group-based ACT on cognitive strategies used by patients with CVDs for emotion regulation and self-control.	Hayes’ ACT for EG, no intervention for CG.	The treatment increased positive coping strategies and decreased negative ones in cardiovascular patients, but self-control did not show significant changes.	Wilks’ Lambda F(3,23) = 85.3, *p* = 0.023, η^2^ = 0.33, power = 0.75; Positive ER *p* = 0.004, η^2^ = 0.29, power = 0.86; Negative ER *p* = 0.045, η^2^ = 0.15; Self-control n.s.
Fattahi A et al., 2023 [40]	Randomized clinical trial	40 CVD patients randomly divided into EG and CG.	Examine how ACT affects distress, emotion regulation, and self-compassion in CVDs patients.	ACT for EG, cardiovascular drugs for CG	ACT decreased depression, anxiety, and stress while boosting self-compassion and emotion regulation.	Mixed ANOVA: significant effects for all variables (*p* < 0.05); effect strongest for stress; η^2^ and power NR
Large R et al., 2020 [41]	Qualitative	13 post-stroke participants	Investigate how ACT can help survivors coping with residual stroke symptoms.	Didactic ACT group sessions.	ACT helped survivors accept symptoms and adjust to limitations. It has shown potential as a psychological intervention for those experiencing distress.	Qualitative study; no statistical data applicable; 6 themes identified (e.g., acceptance, peer support, emotional flexibility)

Effect sizes and power analysis results are reported as available.

**Table 2:** Characteristics of ACT interventions in included studies.

Study	Country	Delivery Mode	Number of Sessions	Session Duration	Therapist Qualifications
Al-Hammouri et al. (2020) [37]	Jordan	Not reported	Not reported	Not reported	Not reported
Ghahnaviyeh et al. (2020) [38]	Iran	Face-to-face	8	90 min	Clinical Psychologist
Sheibani et al. (2019) [39]	Iran	Group-based, face-to-face	8	90 min	Not reported
Fattahi et al. (2023) [40]	Iran	Group-based, face-to-face	8 weekly sessions	Not specified	Clinical Psychologist
Large et al. (2020) [41]	UK	Group-based, face-to-face	8	Not reported	Chartered Clinical Psychologist

When not provided in the original article, ‘Not reported by authors’ is indicated.

## Data Availability

The data that support the findings of this study are available from the corresponding author, upon reasonable request.

## Supplementary Materials

The following supporting information can be downloaded at https://www.mdpi.com/article/10.3390/healthcare13151831/s1, PRISMA 2020 Checklist. Ref. [51] is cited in the Supplementary Materials.
